# Evidence base for patient and public involvement in clinical trials (EPIC)

**DOI:** 10.1186/1745-6215-14-S1-O34

**Published:** 2013-11-29

**Authors:** Carrol Gamble, Louise Dudley, Jennifer Newman

**Affiliations:** 1 University of Liverpool, Liverpool, UK

## Background

Patient and public involvement (PPI), inclusion of members of the public as active partners in the research process, can be a requirement of research funding. Limited evidence has suggested that clinical trials may be particularly likely to benefit from PPI. There is a lack of critical assessment of PPI and suggestions in the literature of selective reporting of its benefits. This study aims to increase the knowledge of PPI in clinical trials by systematically investigating how it is approached within a cohort of public funded randomised trials in the UK.

## Methods

Documentation from the application process for all randomised trials funded by the National Institute for Health Research Health Technology Assessment (NIHR HTA) programme between 2006 and 2010 were obtained. Data relating to PPI activity and how these plans were assessed within the peer review process were extracted.

## Results

Documentation to allow details of planned PPI activity to be extracted were available in 107 RCTs. PPI representation was not described in 15% with representation on TSCs, DMCs, and TMGs described in 53%, 8%, and 12% of applications respectively. A consultancy approach was described in 26%.

Of the 515 reviewers comments across the trials only 211(41%) commented on PPI plans. The majority repeated the PPI plans from the application rather than commenting on their suitability.

## Conclusions

Research applicants frequently describe PPI but reviewers seem unable to comment on suitability of the approach. Evidence of impact is needed to inform future approaches for applicants and peer reviewers.

